# Reproductive biology of the deep brooding coral *Seriatopora hystrix*: Implications for shallow reef recovery

**DOI:** 10.1371/journal.pone.0177034

**Published:** 2017-05-16

**Authors:** Rian Prasetia, Frederic Sinniger, Kaito Hashizume, Saki Harii

**Affiliations:** Sesoko Station, Tropical Biosphere Research Center, University of the Ryukyus, Motobu, Japan; Academia Sinica, TAIWAN

## Abstract

Mesophotic coral ecosystems (MCEs, between 30 and 150 m depth) are hypothesized to contribute to the recovery of degraded shallow reefs through sexually produced larvae (referred to as Deep Reef Refuge Hypothesis). In Okinawa, Japan, the brooder coral *Seriatopora hystrix* was reported to be locally extinct in a shallow reef while it was found abundant at a MCE nearby. In this context, *S*. *hystrix* represents a key model to test the Deep Reef Refuge Hypothesis and to understand the potential contribution of mesophotic corals to shallow coral reef recovery. However, the reproductive biology of mesophotic *S*. *hystrix* and its potential to recolonize shallow reefs is currently unknown. This study reports for the first time, different temporal scales of reproductive periodicity and larval settlement of *S*. *hystrix* from an upper mesophotic reef (40 m depth) in Okinawa. We examined reproductive seasonality, lunar, and circadian periodicity (based on polyp dissection, histology, and *ex situ* planula release observations) and larval settlement rates in the laboratory. Mesophotic *S*. *hystrix* reproduced mainly in July and early August, with a small number of planulae being released at the end of May, June and August. Compared to shallow colonies in the same region, mesophotic *S*. *hystrix* has a 4-month shorter reproductive season, similar circadian periodicity, and smaller planula size. In addition, most of the planulae settled rapidly, limiting larval dispersal potential. The shorter reproductive season and smaller planula size may result from limited energy available for reproduction at deeper depths, while the similar circadian periodicity suggests that this reproductive aspect is not affected by environmental conditions differing with depth. Overall, contribution of mesophotic *S*. *hystrix* to shallow reef rapid recovery appears limited, although they may recruit to shallow reefs through a multistep process over a few generations or through random extreme mixing such as typhoons.

## Introduction

Worldwide coral reefs are strongly threatened by combined ongoing climate changes (i.e. global warming and ocean acidification) and local stressors [1,2]. However, some coral populations may be relatively preserved from these threats such as those found in upwelling, offshore or turbid areas, as well as in deeper parts of the reef due to low exposure to increased sea surface temperature and ultraviolet radiation [3–5]. Recently, greater attention has been paid to the deeper reefs, referred to as Mesophotic Coral Ecosystems (MCEs; below 30 m to over 150 m depth) [6,7], with the assumption that they are buffered from thermal stress [3]. Indeed, some healthy populations of coral species damaged in shallow reefs following bleaching events were found in MCEs [8]. Their role as refuges (at shorter ecological timescales [9]), referred to as the Deep Reef Refuge Hypothesis (DRRH) [3,10,11], was further developed to include their contribution as larval sources for shallow reef recovery [10]. Consequently, the number of studies on MCEs have increased exponentially in the last decade [12]. However, there is still a real gap in the understanding of the functioning and role of the MCEs, especially in their contribution for shallow reef recolonization through vertical connectivity [13]. To date, studies on the sexual reproduction of mesophotic corals have only focused on a very limited number of species [14–20].

Sexual reproduction and larval recruitment are crucial for the maintenance of coral populations. Reproductive mode of corals can be classified as gamete spawners or brooders [21]. Spawning corals release both eggs and sperm into the water column where external fertilization occurs, while brooding corals release larvae, the planulae, directly from the polyps. Reproductive periodicity of corals in MCEs has been studied for both spawning (7 species studied [16,17,19,20]) and brooding corals (2 species studied [15,18]). Previous studies reported that spawning of mesophotic corals was synchronized with their conspecifics living in shallow reefs [16,17,20]. However, currently only few studies have been conducted on brooding corals in MCEs [15,18]. Among these rare study cases, one reports that *Stylophora pistillata* colonies from mesophotic reefs appear to have a shorter reproductive period compared to their conspecifics from shallow reefs [15], yet no studies specifically compared different temporal scales of reproductive periodicity (from seasonal to circadian).

Reproductive periodicity of shallow brooding corals showed complex patterns among species as well as geographic locations [22,23]. Indeed, seasonality of planula release within a species varies depending on the location [24–33]. For example, planula release of three pocilloporid species, *Pocillopora damicornis*, *Seriatopora hystrix*, and *Stylophora pistillata*, showed shorter reproductive period at higher latitudes than at lower latitudes [24,25,27,29] (but see [28,30]). *Porites astreoides* released planulae during two to three months in Bermuda [32], and during five to six months in Florida Keys [33]. The majority of these coral species release their planulae when the seawater reaches relatively high temperatures (> 24.5°C [31–33]). Additionally, within the reproductive season, lunar periodicity of planula release can vary across species within a location as well as within species across locations [31,34,35]. However, the lunar periodicity of planula release in brooding corals seems to constitute a most uncertain environmental predictor compared to the seasonality, suggesting there is no single common environmental cue related to lunar cycle controlling larval release [31,35]. At a circadian timescale, peak of planula release mostly occurs at dawn and dusk [35–37]. The photoperiod has been suggested as a key factor controlling this circadian periodicity [38]. In addition, planula release in the dark may be part of a strategy to reduce the exposition to predation [37,39]. Variations in reproductive periodicity likely correspond to adaptations to local environmental conditions in order to increase success of larval dispersal and recruitment [21]. In this context, corals with broad depth distribution represent useful biological models to assess the environmental cues that are determinant to regulate reproductive periodicity.

*Seriatopora hystrix* is a widespread scleractinian coral [40] and can be found from shallow to mesophotic depths [41–43]. It is known to be a hermaphroditic brooding coral, which releases planulae already associated with symbiotic algae [23,26]. This species produces planulae both sexually and asexually [44–46], although the precise nature of this process is poorly known. Planula release of this species has been widely investigated in several geographic locations, but only from shallow reefs (< 15 m depth) [26,30,31,35–38,47,48]. In Okinawa, *S*. *hystrix* has been found abundant in a MCE [8], while this species was reported to be locally extinct in a shallow reef nearby, in consequence of severe bleaching events in 1998 and 2001 [49,50]. Therefore, *S*. *hystrix* represents an ideal model to assess the potential role of MCEs for shallow reef recovery through the recruitment of larvae. Here we report on different temporal scales of reproductive periodicity in mesophotic *S*. *hystrix* as well as subsequent settlement of released planulae; both being biological processes essential to test the validity of the DRRH hypothesis.

## Materials and methods

### Study site

Samples of *S*. *hystrix* were collected by SCUBA diving from an upper MCE (38–42 m depth) off Sesoko Island, north Okinawa, Japan (26° 40.2' N, 127° 51.9' E). Coral colonies were sampled under permits issued by Okinawa Prefecture Office, Japan (No. 25–18, 26–26, 27–28 and 28–21 from 2013 to 2016, respectively). This MCE was selected for its high abundance of *S*. *hystrix* between 35 to 47 m [8]. Only 7.7 to 9.7% of light measured at the surface penetrated to the study site [20] which is also characterised by a relatively high coral diversity compared to other MCEs currently known in the Ryukyu Archipelago [51].

### Polyp dissection and histology

Fragments (10–15 cm long) were collected from five to ten *S*. *hystrix* colonies (> 15 cm diameter; 5 to 7 m apart) monthly between January 2013 and July 2015 for polyp dissection and histological analyses. During the expected reproductive period, samples were collected up to four times per month. Samples were not collected in February and March 2013 and 2014, and from September 2014 to March 2015 due to bad weather conditions and logistical constraints. In laboratory, fragments were fixed and decalcified using Bouin’s solution for at least 2–4 days. After decalcification, the samples were preserved in 70% ethanol until polyp dissection and histological observations. Only parts approximately 2 to 3 cm below the branch tip were dissected to avoid sterile areas of the branches. In total, five to six fragments per colony, containing each 25–30 polyps, were dissected under a dissecting microscope in order to record the presence of planulae. In complement, histological examination was conducted in order to clarify the presence of planulae in the polyp. Two to five individual polyps per colony predicted to contain planulae were selected, then dehydrated in series of ethanol and xylene solutions, embedded in paraffin, and cut longitudinally in 5 μm-thick sections. The tissues were stained with Delafield’s hematoxylin and eosin before observation under an optical microscope.

### *Ex situ* observation of planula release

Six to ten intact or partial colonies (> 15 cm diameter) were collected every month in 2013 (July and August), 2015 (April to July) and 2016 (May to August). Additional colonies were added and some infertile colonies (confirmed by polyp dissection) were returned back to their native reef in order to ensure planula presence at least in 6 colonies. They were directly transported to Sesoko Station (University of the Ryukyus) using sealed black containers to avoid light stress. Upon arrival at the laboratory, each colony was isolated in a plastic translucent bucket (30 cm in diameter x 14 cm in height) containing around 10 L of 0.2 μm-filtered seawater (FSW). In 2013 and 2015, colonies were maintained in indoor closed seawater systems set with 13 h:11 h (light:dark) photoperiod using LED lights (light intensity maximum 50–60 μmol quanta m^-2^ s^-1^) and with controlled room temperatures (24 ± 1°C in May to June and 27 ± 1°C in July and August). Between 50 and 60% of FSW was renewed two times a day. In 2016, colonies were maintained in indoor running seawater systems with similar light system as described above and the temperatures were controlled using two seawater chillers. To avoid the planulae escaping from the bucket in the running seawater systems, nylon screens (100 μm mesh) were set at the water outlet. Fluorescence of planulae was detected by using a blue-light torch and yellow-barrier filter to examine their potential presence inside mature colonies.

To examine reproductive seasonality, larval release of collected colonies was observed daily from July 18 to August 30, 2013, June 10 to September 14, 2015 and from May 16 to September 10, 2016. For lunar periodicity, the colonies were monitored daily for 2 lunar cycles in 2015 (June 16 to August 11) and in 2016 (July 4 to August 30). Planulae were collected and counted visually using a pasteur pipette. Number of planula release was log (p+1)-transformed to reduce high variability of data. In order to observe circadian periodicity of planula release, three colonies were monitored every 8 h during 3 consecutive days (July 1 to 3, 2015) during the period of abundant planula release.

### Larval size and settlement

The size of the planulae was measured in July 2013 (n = 25 from 9 colonies; 1–5 individuals per colony) and in July and August 2015 (n = 44 from 6 colonies; 2–16 individuals per colony). Planula size was estimated by measuring their maximum length (l) and maximum width (w) under a dissecting microscope. Assuming that the planula shape is a spheroid [52], their volume (V) was calculated using the following equation: V = π/6 · l · w^2^.

In July 2013, planulae from 3 colonies were collected and used for the settlement experiment. Thirty to fifty newly released planulae (from 3 colonies) were transferred to each of 12 replicate plastic containers (volume 500 ml) filled with FSW. The limestone settlement tiles (5 cm x 5 cm x 0.5 cm) were set about 1 cm above the bottom of the containers to facilitate larval settlement. The larvae were counted over 6 days (12, 24, 48, 72, 96, 120, and 144 hours after larvae released) and were classified as (i) swimming or crawling, (ii) metamorphosed or settled on tiles, (iii) metamorphosed or settled on plastic jars, or (iv) dead. About 50–60% of FSW was renewed twice a day.

### Seawater temperature

A temperature logger (HOBO Pro v2, Onset Computer Corporation, USA) was attached to a rope and set to measure at every 5 m depth from surface to 40 m depth on each sampling day from January 2013 to July 2014. In addition, temperature loggers were set to measure hourly and deployed at the sampling site (40 m depth) from April 2014 to August 2016 and at 1–2 m depth on the reef flat in front of the station from January 2013 to August 2016.

### Statistical analyses

The assumptions of normality and homogeneity were verified with Shapiro-Wilk and Levene’s tests, respectively, for each set of data (i.e. circadian periodicity, planula volumes, and settlement preferences) transformed if necessary (log, inverse sine and square root transformation). Parametric tests were used when the data met these assumptions, while non-parametric tests were used when the data did not meet these assumptions. For the circadian periodicity, the effect of time (every 8 h monitoring period) for larval release was analyzed using Kruskal-Wallis`test and then Dunn’s test for *post hoc* comparisons. An independent *t*-test was used to compare planula volume between years. A Mann-Whitney U test was used to compare percentages of larvae settled on different substrate materials after 12 hours. The significance level was set as 0.05 in all tests. All statistical analyses were conducted using R software [53].

## Results

### Seasonality of planula release

**Fig 1:**
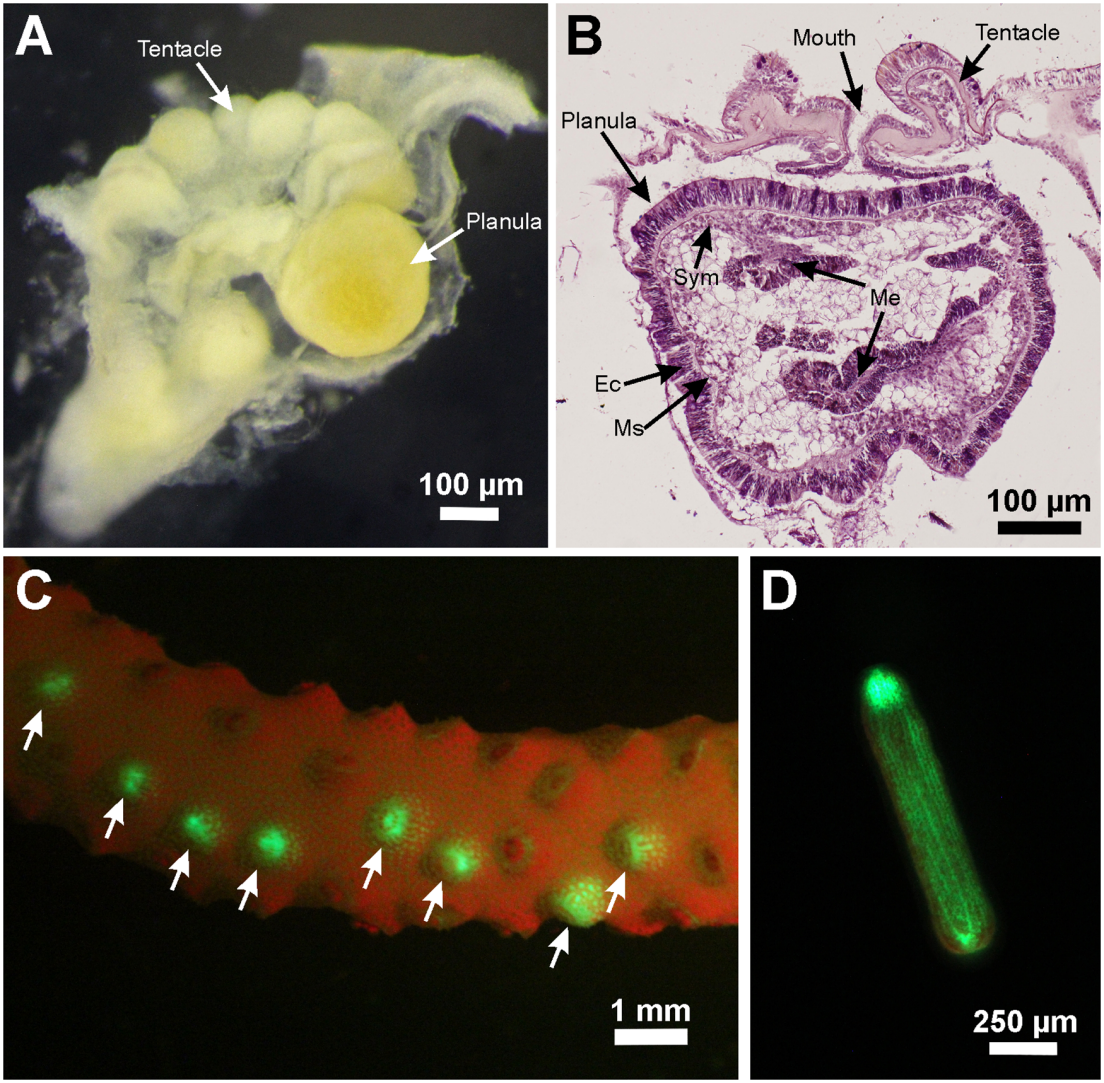
Planulae from polyp dissection, histology, and planula release observation. (A) Dissected and (B) longitudinal section of a polyp (July 18, 2013). (C) Planula larvae (pointed arrows) inside a fragment (June 29, 2015) and (D) a planula larva containing green fluorescence protein (July 4, 2015). Labels: *Ec* ectoderm, *Ms* mesoglea, *Me* mesentery, *Sym Symbiodinium* sp.

**Fig 2:**
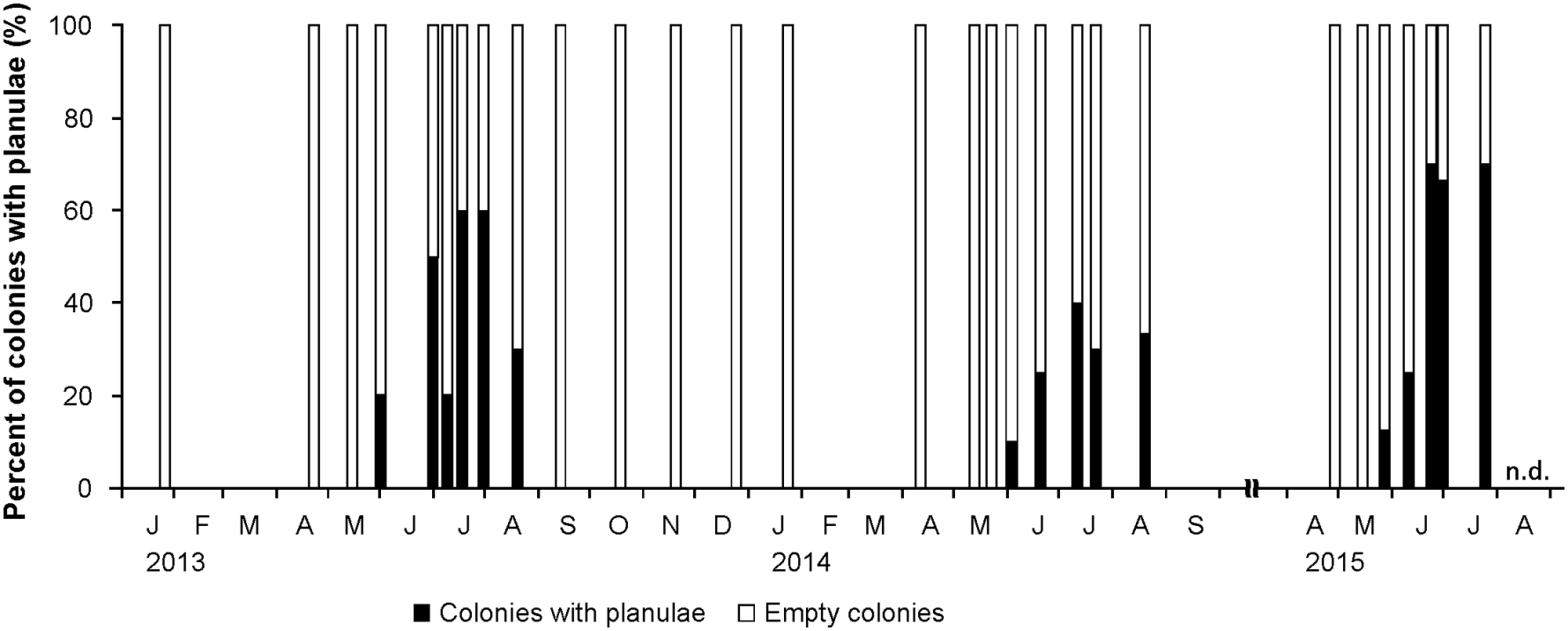
Temporal changes in the proportion of colonies containing planulae of *Seriatopora hystrix*. Five to ten colonies were observed through polyp dissection and histology at each sampling date. nd = no data.

During the 3-year monitoring (2013–2015), planulae of mesophotic *S*. *hystrix* were observed in the polyps (Fig 1A and 1B) from May to August, and non-existent in other months (Fig 2). Planulae were first observed in the polyps at the end of May, with planulae present in 12.5–20% of the colonies. Peak planula production occurred in June to July, with 10–70% of colonies containing planulae. In August, planula production decreased with 30–33% of colonies containing planulae (Fig 2).

**Fig 3:**
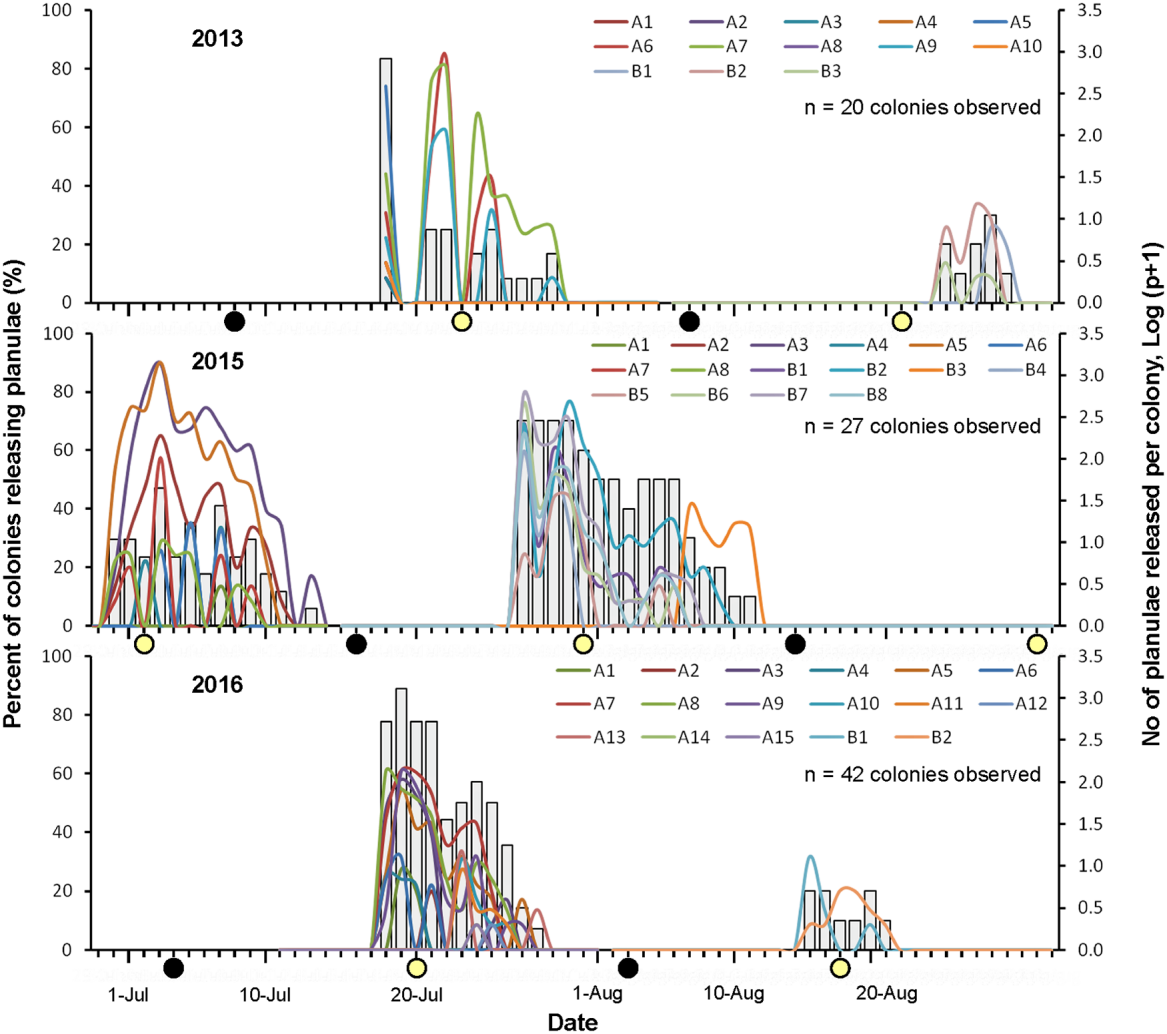
Temporal change of *Seriatopora hystrix* planula release. The data is shown as percent of colonies releasing planulae (bar graphs) and number (log p+1-transformed) of planulae released (line graphs) of each colony between June 29 and August 31 (2013, 2015, 2016). The alphabet-number combination (e.g. A1, B1) represent lunar (A, first and B, second lunar cycles)-individual colony number that released planulae. New moon (black circles) and full moon (yellow circles) phases are plotted in the graphs.

Planula release occurred mainly between July and early August, with up to 83, 70, and 90% of colonies releasing planulae in 2013, 2015, and 2016, respectively (Fig 3). During these periods, planula release lasted 9 (2013), 28 (2015) and 11 (2016) days (Fig 3). A small proportion of colonies (10–30%) additionally released planulae in late August 2013 and 2016 (Fig 3). At the end of August, the release period extended only for 5 to 6 days (Fig 3). In addition, three planulae were released from a small fragment sampled at the end of May 2013 for polyp dissection and histological observations, however it is not clear whether this release was induced by sampling stress and thus this result was not considered further.

### Lunar and circadian periodicity of planula release

In 2015 and 2016, the colonies started to release planulae between lunar day 12 and 15 (around full moon; lunar day 1 = new moon). They released planulae until lunar day 25 to 28 (third lunar quarter), except in August 2016 when the colonies released planulae until lunar day 18 (Fig 3). The average duration of planula release for a colony was 6.0 ± 0.7 days (mean ± SE, range 1–14, n = 33). In 2013, the lunar pattern could not be summarized since the colonies were not observed for a complete lunar cycle. In 2013, 2015 and 2016, the total numbers of planulae released were 2953, 11507 and 1617, respectively. However, in each year, most of the planulae were released from 4 to 6 colonies, while the other colonies released less than 100 individuals per colony within a lunar cycle.

**Fig 4:**
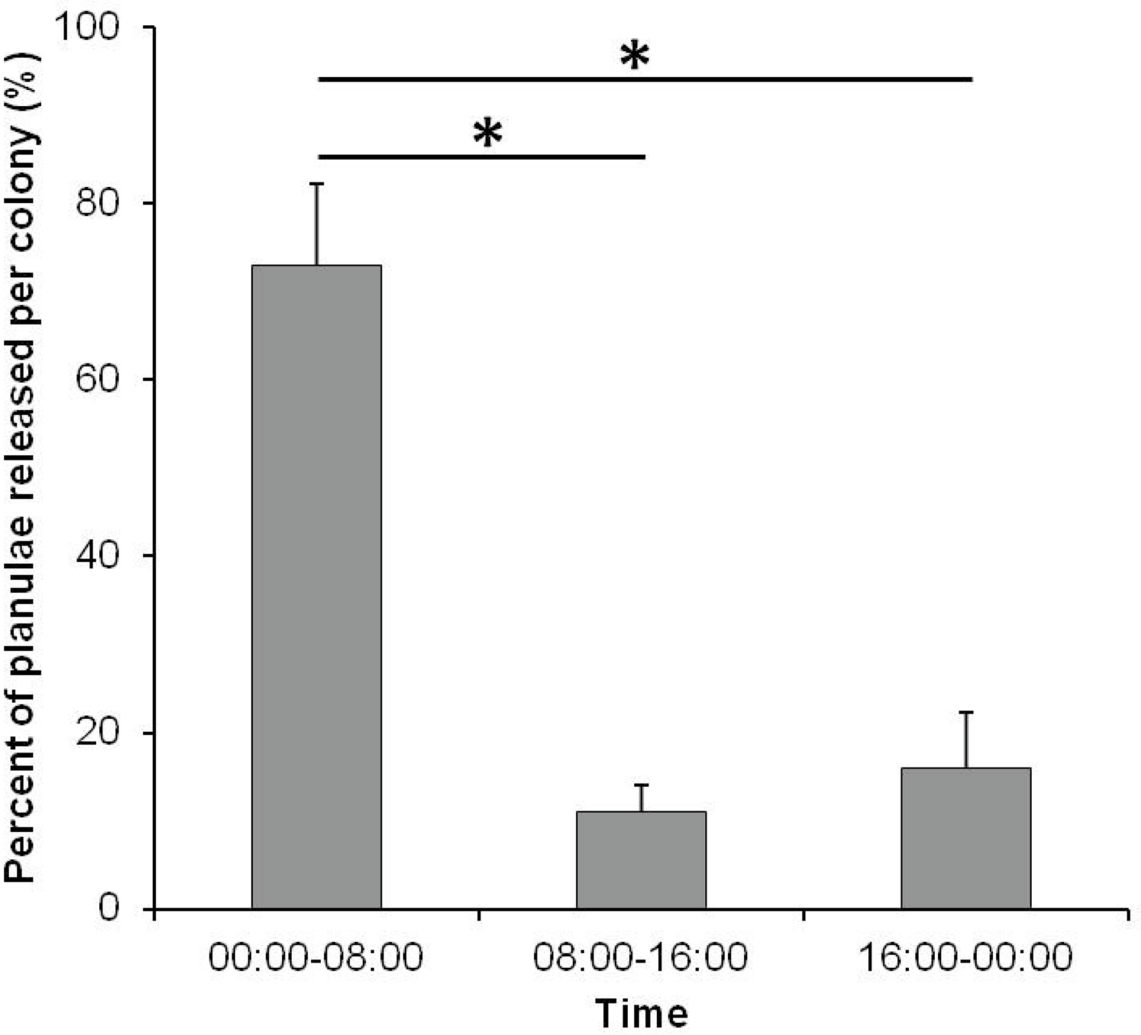
Circadian pattern of planula release of *Seriatopora hystrix*. The data is shown as percentage of total planulae released during the 8 h monitoring intervals within the circadian cycle (24 h) per colony from July 1 to 3, 2015 (mean ± SE, n = 9). Significantly different comparisons are indicated with an asterisk.

The circadian periodicity of planula release of *S*. *hystrix* was highly consistent and synchronized occurring from midnight to 8:00 AM (Fig 4, S1 Table). During these hours, 72.9 ± 9.2% of the planulae (mean ± SE, n = 9) were released. A low percentage of planulae were still released from morning until midnight.

### Characteristics and settlement of planulae

**Fig 5:**
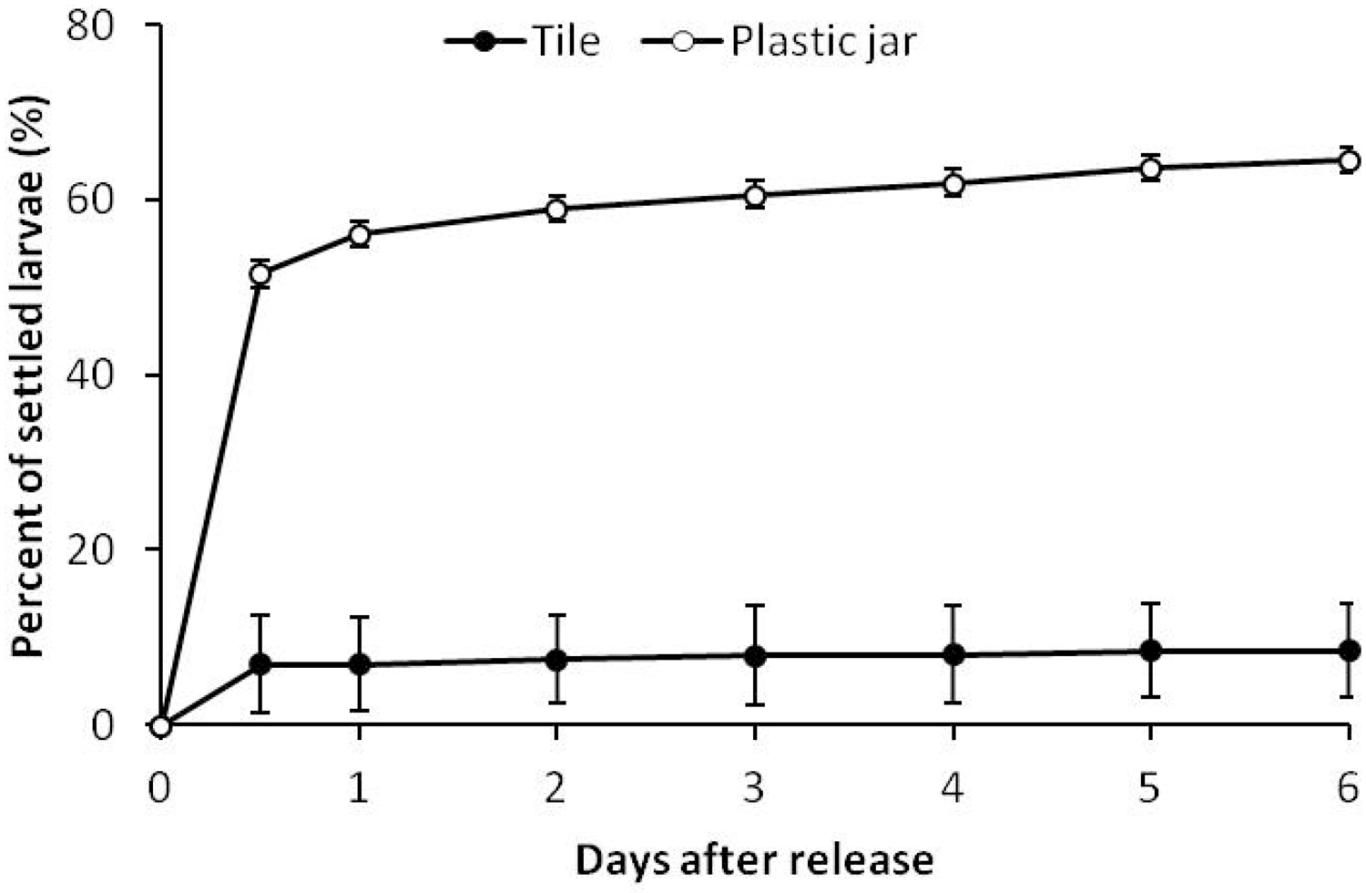
Percentage of *Seriatopora hystrix* planulae that settled on tiles and plastic jars over 6 days of observation after planula release (mean ± SE, n = 12).

The planulae of *S*. *hystrix* were deep brown due to the presence of *Symbiodinium* symbiotic algae. Under blue light, they also showed green fluorescence likely from green fluorescence protein (Fig 1C and 1D). They were typically rod or pear-shaped. The volume of planulae did not differ significantly between years (S1 Table), with mean volume of 0.05 ± 0.004 and 0.06 ± 0.003 mm^3^, in 2013 and 2015, respectively (± SE; 2013, n = 25; 2015, n = 44). After release, the planulae were actively crawling at the bottom and side of plastic jars and they were competent to settle immediately after being released. Within 12 hours, 6.9 ± 1.5% and 51.6 ± 5.6% (mean ± SE, n = 12) of planulae settled both on the tiles and on the plastic jars, respectively (Fig 5). In all monitoring periods, most of the planulae preferred to settle on the plastic jars (Fig 5, S1 Table).

### Seawater temperature

**Fig 6:**
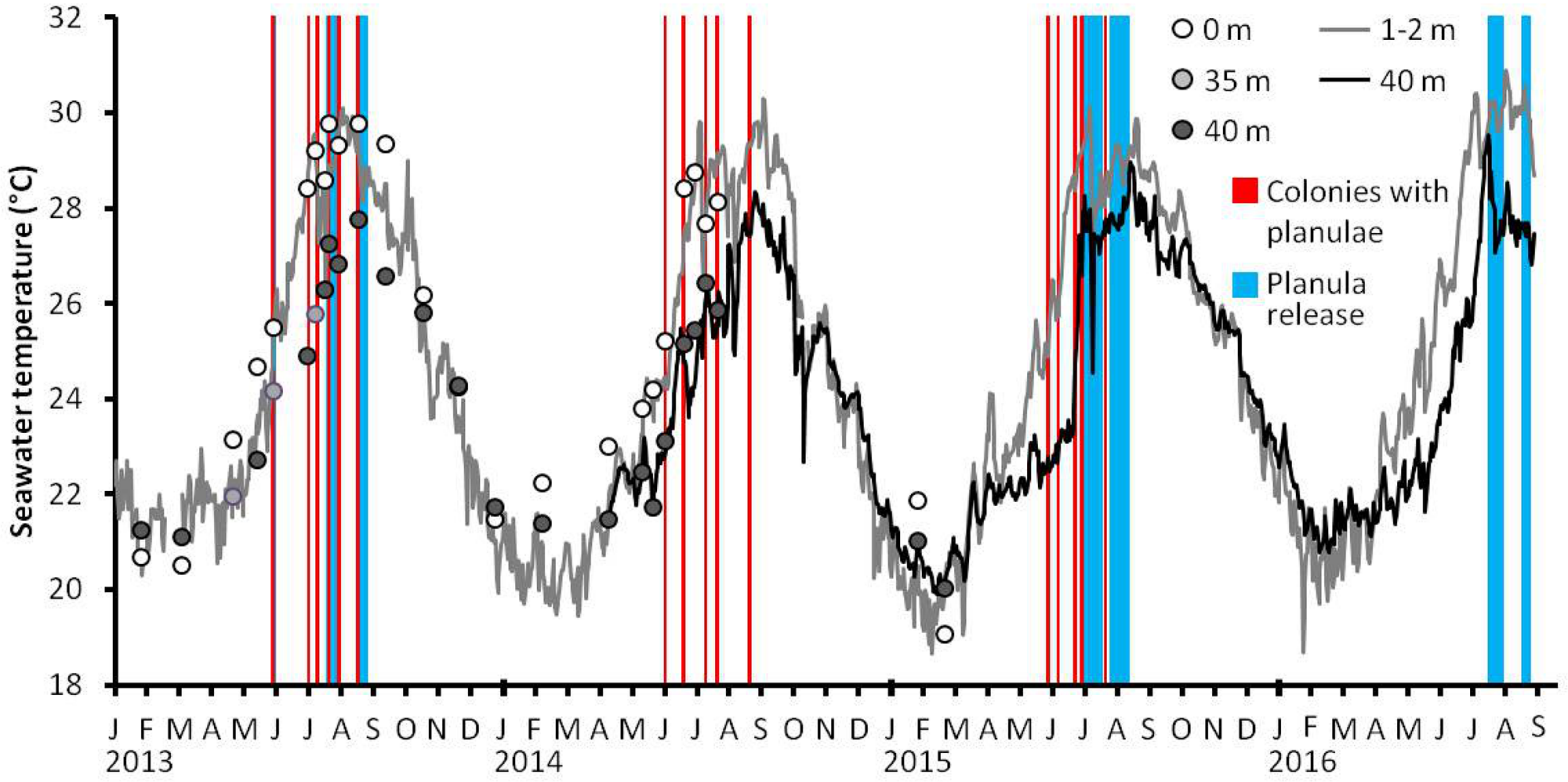
Mean daily seawater temperature from surface to 40 m depth between January 2013 and August 2016. Seawater temperatures at surface (white circles), 35 m depths (light grey circles), and 40 m depths (dark grey circles) off Sesoko Island were recorded between January 2013 and July 2014. Seawater temperature at a shallow reef (1–2 m) at southern Sesoko Island was recorded between January 2013 and August 2016 (grey lines), while at study site (40 m) was recorded between April 2014 and August 2016 (black lines). The periods when colonies contained (red bars) and released planulae (blue bars) are plotted on the graph.

The maximum daily seawater temperature at 1–2 m was recorded between July and September, while at 40 m it was usually recorded in August, except in 2016 when the maximum seawater temperature at 40 m occurred in the middle of July (Fig 6). The minimum seawater temperature was recorded between January and March at both depths. The annual seawater temperature ranges at 40 m is narrower compared to the seawater temperature at 1–2 m. During the planula release period (Fig 3), the minimum seawater temperature at 35–40 m depths was 24.2, 24.6, and 27.1°C recorded at the end of May 2013, July 2015, and July 2016, respectively (Fig 6). During the major planula release between July and early August, seawater temperature ranged between 24.2–27.3, 24.6–28.3, and 27.1–28.8°C in 2013, 2015, and 2016, respectively (Fig 6).

## Discussion

Planula release of mesophotic *S*. *hystrix* in Okinawa mainly lasted from July to early August. This corresponds to a 4-month shorter reproductive season compared to shallow *S*. *hystrix* which release planulae from May to October nearby [48]. A shorter planula release period for deeper colonies has also been suggested for another brooding coral species in the Red Sea [15]. Deeper colonies (25–42 m depth) of *Stylophora pistillata* were estimated to have a 2–3 month shorter reproductive season compared to their conspecific colonies in the shallow reefs (3–9 m depth). Rinkevich and Loya suggested that deep *S*. *pistillata* receive limited energy supply from photosynthesis for reproduction [15]. Indeed, low light availability was later shown to minimize the energy allocation from symbionts for gametogenesis in corals [54]. Since brooding corals have multiple larval release periods per year, low light intensity (~10% light availability compared to the surface, [20]) likely reduces their number in mesophotic *S*. *hystrix* in Okinawa.

Seawater temperature may also play a role in the reproductive seasonality of mesophotic *S*. *hystrix*. In our study planula release occurred only when seawater temperature was > 24.0°C, which was also observed in shallow conspecific colonies in the region [48]. It is congruent with the knowledge that the maturation and release of gametes are related to warm temperature [55–58]. Indeed, the period of planula release of shallow *S*. *hystrix* is shorter in high latitudes than low latitudes; it occurs for 12 months at lower latitude in Palau (7° N), while it occurs for 8 months in Taiwan (21° N) and 6 months in Okinawa (26° N) (Table 1). In the southern hemisphere, at latitude comparable to Taiwan, shallow *S*. *hystrix* releases larvae for 8 months in the GBR (23° S) (Table 1). In addition, in our study, only a low percentage of colonies released planulae at the end of August when the peaks of seawater temperature were 27.8°C and 29.0°C in 2013 and 2015, respectively. In addition, low amounts of planulae were released in 2016 when seawater temperature was above 29.0°C in the middle of July. Thus, excessively high temperature might inhibit gamete maturation and subsequent planula development in July and/or August, explaining the absence of planula presence in the following months. Similarly, high seawater temperature in Bermuda has been shown to reduce the number of planulae released from *Porites astreoides* [32] and seawater temperature outside the optimum temperature for reproduction has been shown to significantly reduce reproductive output of *Pocillopora damicornis* [59]. Overall, short reproductive season of *S*. *hystrix* in MCEs is controlled by a combination of limited light and short period of optimum seawater temperature for reproduction.

**Table 1:** Seasonality, lunar and circadian periodicity of larval release of *Seriatopora hystrix* recorded from different geographic locations and different depths. Data are ordered by latitudes from North to South. (-) indicates no available data. GBR: Great Barrier Reef. ^a^ No depth information; depth assumed as shallow. ^b^ Unpublished data from the same study suggest planula presence in the polyps throughout the year.

Site	Latitude	Annual temperature range (monthly averages)	Depth (m)	Planula release			Sources
				Seasonality	Lunar periodicity	Circadian pattern	
Okinawa	26° N	7.3°C (20.3–27.6°C)*	40	July to Aug	Full moon to 3^rd^ quarter	00:00 to 08:00 am	**This Study**
		8.6°C (20.4–29.0°C)*	Shallow^a^	May to Oct	New to Full moon	-	[48]
			Shallow^a^	-	-	03:00 to 05:00 am	[36]
Taiwan	21° N	7°C (22.5–29.5°C)	0–15	Jan to May; Sep to Nov ^b^	All period	-	[30]
			Shallow^a^	-	New moon to 1^st^ quarter	-	[60]
			3–8	-	-	Close to sunrise	[37]
			3–10	-	-	Close to sunrise	[38]
Philippines	16° N	3.1°C (26.7–29.8°C)**	4–7	-	3^rd^ quarter to New moon	-	[35]
Enewetak	11° N	2.5°C (26.7–29.2°C)**	0–2	-	All period	-	[47]
Palau	7° N	2.4°C (27.4–29.8°C)**	Shallow^a^	All months	All period	-	[26]
GBR	23° S	10 °C (19–29°C)***	5–10	Sep to May	1^st^ quarter to Full moon	-	[31]

Seawater temperature data sources,

*: data in situ;

**:[61];

***: [62]

The lunar periodicity of planula release in mesophotic *S*. *hystrix* differed from shallow conspecific colonies nearby [48], as well as from shallow colonies in different geographic locations (Table 1). In the present study, mesophotic *S*. *hystrix* released their planulae mainly around full moon and up to third quarter moon, while shallow conspecific colonies in the region released their planulae between the new and full moon [48]. In other shallow reef locations, planula release occurred either in all moon phases [26,30,47] or in some specific moon phases [31,35,60] (Table 1). Thus, there is no conserved pattern of planula release following the moon phase for *S*. *hystrix* among different geographic locations. Variability of reproductive periodicity within a lunar cycle was also reported for other brooding coral species (e.g. *Pocillopora damicornis*, *Stylophora pistillata*) in different geographic locations [27,31,35]. The factors influencing lunar periodicity of planula release remain unclear. Lunar periodicity of planula release might be determined by night irradiance [63]. For example, Jokiel et al. [63] demonstrated that *P*. *damicornis* lost their planula release synchronization after being exposed to shifted-phase treatment of lunar irradiance as low as 0.01 μmol quanta m^-2^ s^-1^. However, in our study, such low irradiance might be highly attenuated, and indeed could not be detected by light meters deployed at 40 m depth even during full moon phases (pers. obs.). For brooding corals, lunar periodicity of planula release may be related to the earlier gametogenic phases rather than to cues for planula release itself [64,65]. Specific tidal phases have also been suggested as a cue for the release of male gametes [31]. Thus tidal cycles may explain the lunar synchrony in mesophotic corals, even though our tanks did not reproduce tidal patterns. However, as the monitored colonies were mostly collected during the lunar cycle directly preceding our observations, the male gametes release timing was not affected by our tank conditions, and thus lunar synchrony was still observable in our experiments. In addition, following the release of male gametes, the duration of fertilization and planula development was proposed to explain unsynchronized planula release in brooding corals [66] and this explanation could also explain the differences in duration of planula release in our experiments.

Circadian periodicity of planula release observed here is similar to shallow conspecific colonies in Okinawa, and in different geographic locations (Table 1). In this study, the peak of planula release of *S*. *hystrix* from mesophotic depth occurred from midnight to morning with most planulae being released around 1 to 3 hours before sunrise (pers. obs.). This pattern is similar to shallow *S*. *hystrix* [36–38] as well as to other brooding pocilloporids, such as *Stylophora pistillata* [36,37] and *P*. *damicornis* [35,36]. Lin et al. [38] suggested the light-dark period to be a cue for circadian periodicity of planula release in *S*. *hystrix*. In their experiments, the *S*. *hystrix* colonies did not release planulae during continuous light or continuous dark conditions, and the peak of planula release was cued by sunrise on the previous day. Furthermore, releasing planulae at dawn could be a strategy to avoid visual predators [37,39]. In shallow reefs, some planktivorous fish feed on sperm-egg bundles when light is still sufficient for visual feeding [39,67]. Although nothing is known on the predation of coral planulae at mesophotic depth, this explanation may also apply to *S*. *hystrix* in this study as planktivorous fish species also inhabit MCEs [7,68–70]. The absence of difference with shallow corals suggests that this circadian periodicity is not affected by parameters differing with depth.

Mesophotic *S*. *hystrix* had relatively smaller larval size compared to shallow colonies in Palau [26] and in the same region [36]. For example, the mean volume of planulae in our study was between 1.3 and 5 times smaller than planulae from shallow colonies reported in previous studies [26,36]. Smaller larval size of deep *S*. *hystrix* could result from the low reproductive energy allocation of photosynthetic products in low light environment [71] (but see [17]). Smaller larval size has been related to lower survival and shorter dispersal potential [36]. For example, small-sized planulae of three pocilloporids had short lifetime which could reflect an adaptive strategy to settle on their natal reefs [36]. Marshall and Keough [72] in their review reported that for most lecithotrophic (non-feeding) larvae of marine invertebrates, large-size of egg/larvae was positively related to larval lifespan through providing higher energy reserve. In case of mesophotic corals, reduced light in MCEs might result in small-sized planulae, and may therefore limit the dispersal potential compared to larvae from shallow colonies.

In the present study, the planulae of *S*. *hystrix* preferred to settle on the plastic substrate rather than on the settlement tiles. A similar behavior has been observed in laboratory for shallow *S*. *hystrix* [73], *Acropora millepora* [74] and *Stylophora pistillata* [75]. However, the explanation for this behavior remains unclear [75]. Regardless of substrate preferences, more than half (63%) of larvae from mesophotic depth already settled within 24 hours after being released. This is relatively similar to the settlement rate of larvae from shallow reefs [26,36]. For example, 50–90% and 59% of the planulae already settled within 24 hours after being released for planulae from shallow reefs in Okinawa [36] and Palau [26], respectively. Rapid settlement likely corresponds to relatively high local recruitment, reducing chances for long distance dispersal. This behavior is supported by genetic connectivity studies for shallow *S*. *hystrix*. High level of genetic differentiation suggesting localized recruitment has been reported for this species in GBR [76] (but see [77]), Northwestern Australia [78], and Red Sea [79]. In addition, sperm dispersal of this species has been suggested to be locally restricted, only dispersing within 10 m from their parent colonies [46]. In our study, the short duration of planktonic phase of mesophotic *S*. *hystrix* larvae combined with small planula size and high settlement rate imply that larvae of mesophotic *S*. *hystrix* are more likely to disperse less far than shallow colonies.

The extent to which mesophotic corals serve as sources of larvae for shallow reefs is one of the knowledge gaps remaining to test the validity of the DRRH [10,12,80]. The DRRH implies vertical genetic connectivity between shallow and mesophotic population and sufficient larval exchange from deep to shallow communities [10]. To date, recruitment of mesophotic-originated larvae to shallow reefs is site and species specific and vertical genetic connectivity varies greatly even within a reef [11,43,81,82]. In the case of Okinawa, Sinniger et al. [83] found that no structure related to depth was observed between shallow and mesophotic *Seriatopora* based on mitochondrial and nuclear markers. However, based on our study, direct recruitment of mesophotic *S*. *hystrix* larvae to shallow reefs might be limited since this species has short reproductive season (mainly between July and early August) and the larvae have relatively short dispersal potential (small larval size and quick settlement after release). This pattern supports current knowledge that about 1–10% of mesophotic coral larvae are predicted to recruit to nearby shallow reefs [84]. Larval dispersal and recruitment patterns are largely determined by the larval duration and position in the water column during planktonic phase and the hydrological conditions at the time of floating [21,85]. In the case of mesophotic *S*. *hystrix*, duration of the planktonic phase is short, resulting in a relatively low chance of vertical migration to shallow reefs. However, vertical migration of larvae might be aided by vertical water movement such as from tropical storms [84]. In Okinawa, typhoons frequently pass through during summer months and generate strong water movement in the water column [86]. Therefore, the occurrence of typhoon at the time of larval release could create enough vertical water movement for mesophotic *S*. *hystrix* larvae to potentially reach shallow reefs. However, frequency and paths of strong storms varies between years and this contribution will occur randomly depending on locations and years. Based on all our findings, recolonization of larvae from mesophotic depths to shallow reefs is likely occurring slowly through multigenerational recruitment over a long-term period or through random vertical larval transport by such as strong storms. Furthermore, whether mesophotic coral larvae can adapt to higher seawater temperature and light conditions in shallow reefs after settling remains to be confirmed. Considering the ecological importance of *S*. *hystrix* in Okinawa further investigations are needed to examine its larval behavior and larval acclimation to shallow reef environmental conditions.

## Supporting information

S1 Table Summary of statistical analyses for circadian periodicity of planula release, planula volume between years, and larval settlement in different substrate. Significant differences are shown in bold. Group a, b, and c represent circadian periodicity of planulae release during the following periods: 0:00–8:00 AM, 8:00 AM– 4:00 PM, and 4:00 PM– 0:00 AM, respectively. n.a. = post hoc test was not applicable.
(DOCX)
