# Supplementary material for: Reproductive biology of the deep brooding coral Seriatopora hystrix: Implications for shallow reef recovery
Source: PLoS One. 2017 May 16;12(5):e0177034. doi: 10.1371/journal.pone.0177034 (PMC5433689; doi:10.1371/journal.pone.0177034)
Supplement: S1 Table — Significant differences are shown in bold. Group a, b, and c represent circadian periodicity of planulae release during the following periods: 0:00–8:00 AM, 8:00 AM– 4:00 PM, and 4:00 PM– 0:00 AM, respectively. n.a. = post hoc test was not applicable. (DOCX) [file pone.0177034.s001.docx]

**S1 Table. Summary of statistical analyses for circadian periodicity of planula release, planula volume between years, and larval settlement in different substrate.** Significant differences are shown in bold. Group a, b, and c represent circadian periodicity of planulae release during the following periods: 0:00 – 8:00 AM, 8:00 AM – 4:00 PM, and 4:00 PM – 0:00 AM, respectively. n.a. = post hoc test was not applicable.

| **Kruskal-Wallis** | **d.f.** | **Chi-Square** | ***p*** | **Pairwise comparisons** |
| --- | --- | --- | --- | --- |
| Circadian pattern of planula release | 2 | 14.917 | **< 0.001** | a > (b = c) |
|  |  |  |  |  |
| **Mann-Whitney U** |  |  | ***p*** | **Pairwise comparisons** |
| Larval settlement between substrate type (12 hours) |  |  | **< 0.001** | n.a. |
|  |  |  |  |  |
| **T-test** | **d.f.** |  | ***p*** | **Pairwise comparisons** |
| Volume of planulae between years (i.e. 2013 and 2015) | 48.993 |  | 0.350 | n.a. |
|  |  |  |  |  |
